# Retracted: Maternal traditional Chinese medicine exposure and risk of congenital malformations: A multicenter prospective cohort study

**DOI:** 10.1111/aogs.14649

**Published:** 2023-08-08

## Abstract

The above article, published online on 19 April 2023 in Wiley Online Library (wileyonlinelibrary.com), has been retracted by agreement between the authors, the journal's Editor‐in‐Chief, Ganesh Acharya, and John Wiley & Sons Ltd. The retraction has been agreed due to flaws with the study design in collecting data on exposure to traditional Chinese medicine in pregnant women, which affects the validity of the data to support the conclusions of the study.

**How to cite this article**: Peng, T, Yin, L‐L, Xiong, Y, et al. Retracted: Maternal traditional Chinese medicine exposure and risk of congenital malformations: a multicenter prospective cohort study. *Acta Obstet Gynecol Scand*. 2023;102:735–743. doi:10.1111/aogs.14553
